# Extensive Forehead Swelling Including the Periorbital Area in a Child: A Rare Manifestation of Acute Lymphoblastic Leukemia

**DOI:** 10.7759/cureus.3539

**Published:** 2018-11-03

**Authors:** Maya sapira Hanapi, Siti-Ilyana Ghani, Khairy Shamel Sonny Teo, Wan-Zuhairah Wan-Embong, Nasir Ariffin, Wan-Hazabbah Wan Hitam

**Affiliations:** 1 Ophthalmology, School of Medical Sciences/Universiti Sains Malaysia, Kota Bharu, MYS; 2 Hematology, School of Medical Sciences/Universiti Sains Malaysia, Kota Bharu, MYS; 3 Paediatrics, School of Medical Sciences/Universiti Sains Malaysia, Kota Bharu, MYS

**Keywords:** acute lymphoblastic leukaemia, forehead swelling, evaluation, treatment

## Abstract

Acute lymphoblastic leukemia (ALL) manifestations in a child are varied. We report a unique and rare presentation of acute lymphoblastic leukemia in a child who presented with frontal swelling involving bilateral upper lids. A previously healthy one-year-old girl presented with progressively increasing frontal swelling of seven months duration. An examination revealed erythematous, firm, nontender forehead swelling that extended up to the medial part of bilateral upper eye lids. The extraocular muscle movement was normal. The anterior segment and fundus examination were also normal in both eyes. Other systemic examination revealed multiple leukemic cutis on the scalp. The cervical lymph nodes were also palpable with hepatosplenomegaly. A full blood picture (FBP) showed the presence of leucoerythroblastic blood film with 62% blast cells. Flow cytometry and bone marrow aspiration confirmed the diagnosis. Computed tomographic (CT) scan images revealed multiple well-defined hyperdense lesions at the subcutaneous skull with the largest lesion at the anterior glabella. Upon diagnosis, the patient was started on chemotherapy and the swelling resolved after one month post treatment. Extensive forehead swelling is a rare manifestation of acute lymphoblastic leukemia. A high index of suspicion aided with diagnostic investigations could help the doctors arrive at a correct diagnosis and treatment.

## Introduction

In acute lymphoblastic leukemia (ALL), malignant lymphoid precursors proliferate and replace the normal hematopoietic cells of the marrow, circulate in blood, and invade other tissues of the body [1]. Leukemic cells may infiltrate the skin and the orbit during the course of acute or chronic leukemia. The presentation of leukemic infiltration may vary from asymptomatic and visually-insignificant to severe visual deterioration with motility disturbances. Here, we report a unique and unusual presentation of extensive forehead swelling involving bilateral upper lids in a child with acute lymphoblastic leukemia.

## Case presentation

**Figure 1:**
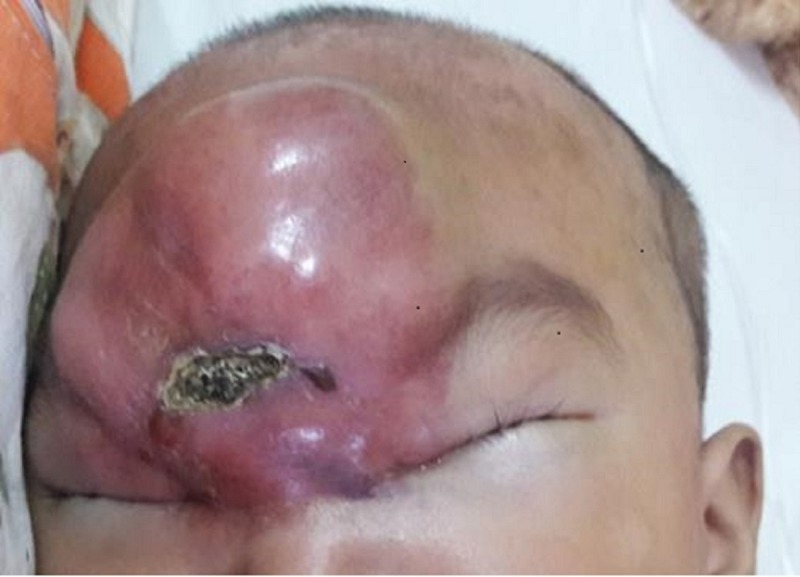
External photo of a child with forehead swelling involving medial side of both upper eyelids with central crusted lesion.

A one-year-old girl presented with progressively increased forehead swelling of seven months duration. The swelling progressively increased in size and extended up to the medial edge of both her upper eyelids (Figure 1). This large swelling caused the patient to have slight difficulties in opening her eyes widely. On the other hand, there was no eye redness, discharge, or reduced vision. The patient also had constitutional symptoms like loss of appetite and weight loss over this period of several months.

On examination, there was a massive, round, multiple lobulated and erythematous swelling extending from the forehead until the medial part of bilateral upper lids with a central crusted lesion surrounded by scaly skin. It was nontender, warm, and firm in consistency. It measured about 7 cm x 7 cm in diameter. Her best corrected vision was 6/6. There was no refractive error noted. Both eyes were orthophoric. The remainder of the eye examination revealed normal anterior segment. The fundus examinations were normal with pink and healthy optic disc. There was no Roth's spot or retinal hemorrhage. The intraocular pressure was normal. The extraocular movements were also normal. A systemic examination revealed multiple leukemic cutis, small red-to-brown lesions on the scalp, and presence of hepatosplenomegaly. Bilateral multiple cervical lymph nodes were palpable. Otherwise, her neurological examination was normal.

**Figure 2:**
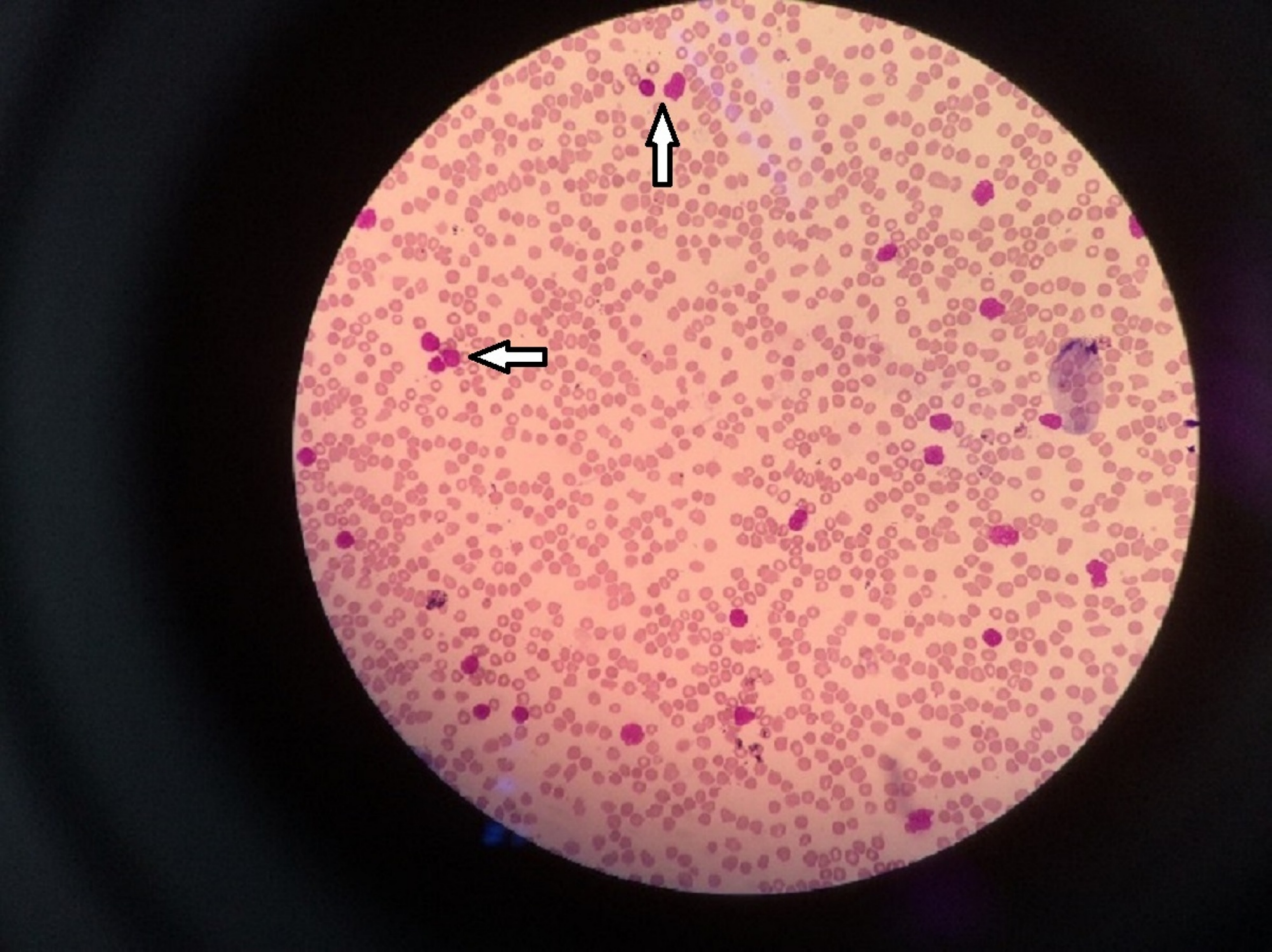
Peripheral full blood picture (FBP) showing presence of numerous blast cells, which are moderate in size with irregular nucleus and scanty cytoplasm.

Her hemoglobin was 9.7 g/dL and the total white cell count (TWBC) was 68.97 x 109/l. Her differential count showed 91.7% lymphocytes, 0.9% neutrophils, and 7.2% monocytes. There was leucoerythroblastic blood film with 62% blast cells on the full blood picture (FBP) (Figure 2).

**Figure 3:**
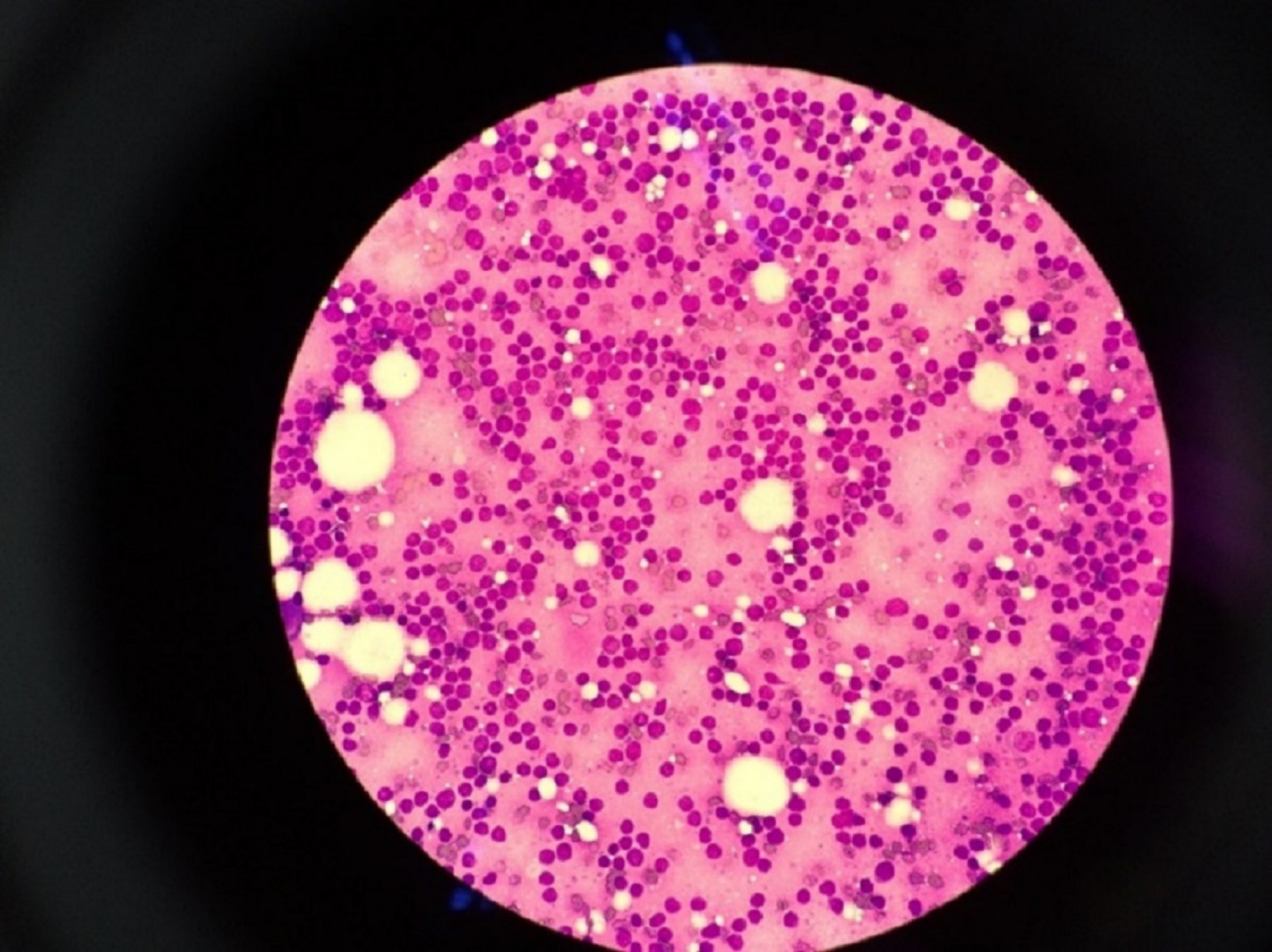
Homogenous population of blast cells from bone marrow trephine imprints.

**Figure 4:**
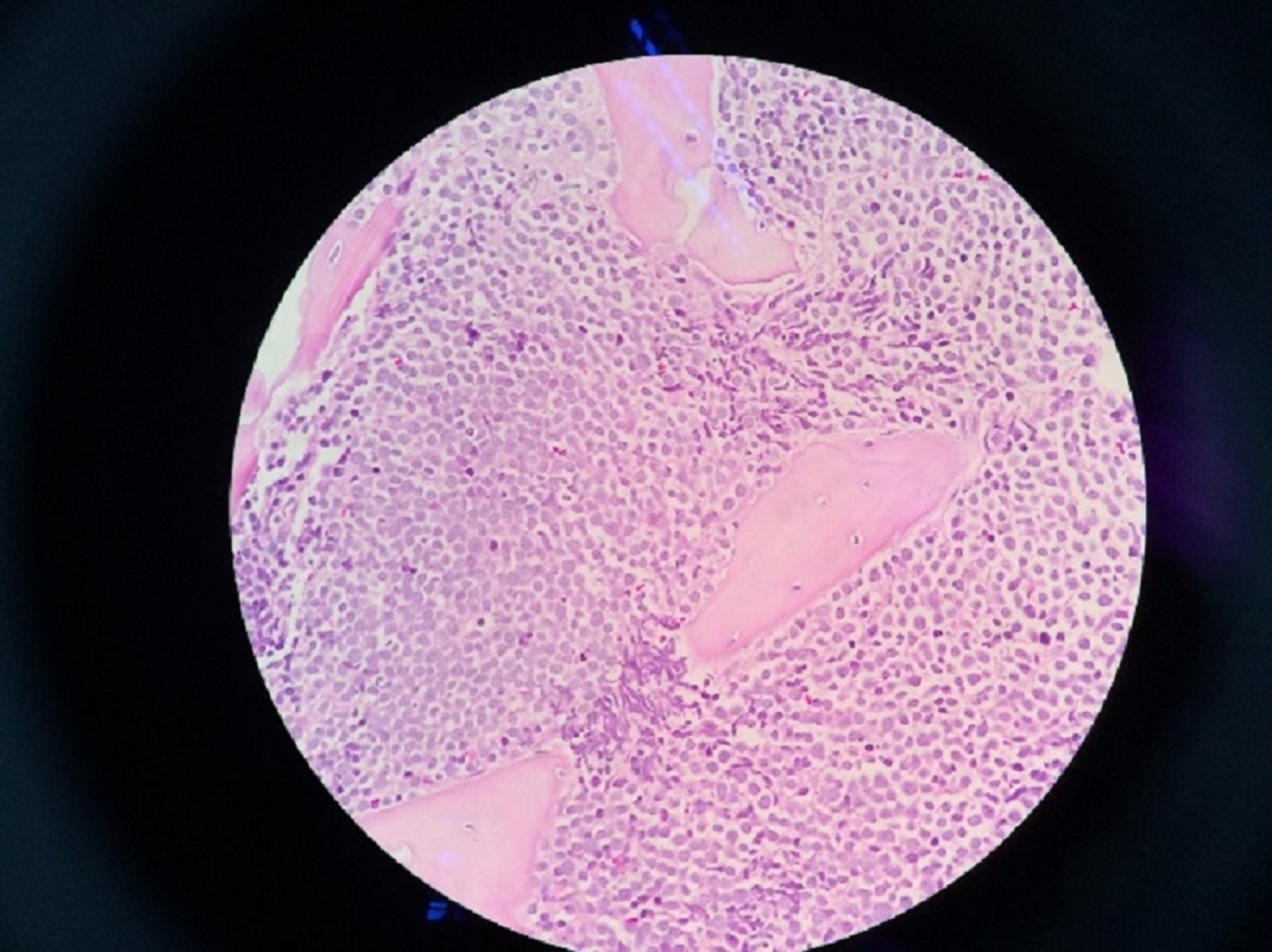
Bone marrow trephine imprint showing blast cells with high nucleocytoplasmic ratio, scanty cytoplasm with some blast cells show prominent nucleoli (magnification: 40X).

Bone marrow aspiration (BMA) showed blast cells, which exhibited high nuclear-cytoplasmic ratio, and scanty cytoplasm with some blast cells showing prominent nucleoli (Figures 3, 4).

**Figure 5:**
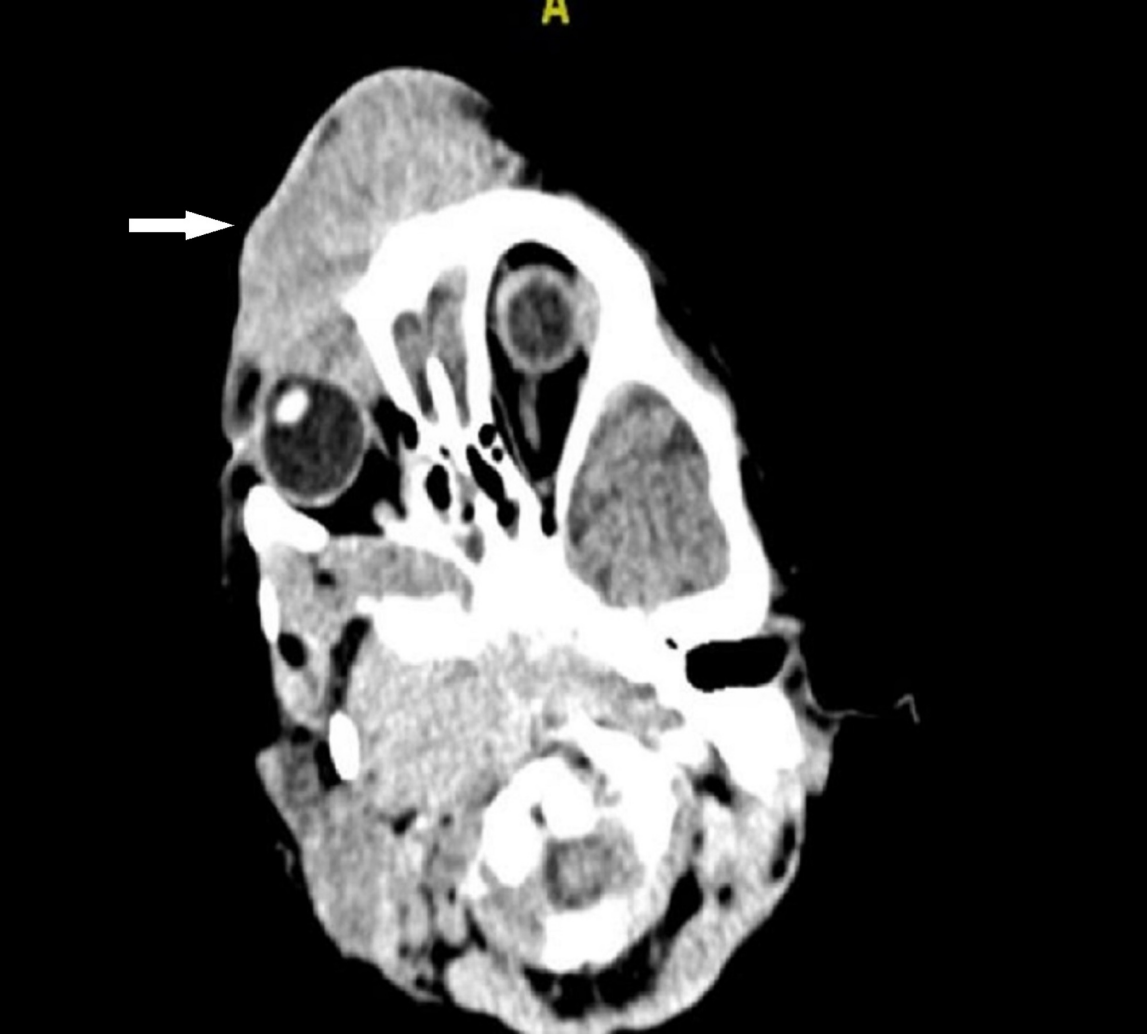
Computed tomography scan of the brain showing multiple well-defined enhanced lesions at the frontal scalp with the largest lesion at the anterior glabella. The lesion extended into the bilateral orbital cavities and caused lateral displacement of the bilateral orbits. The bilateral globes were normal.

The flow cytometry result confirmed the diagnosis of B-acute lymphoblastic leukemia by showing positivity for CD79a, HLA-DR, CD 10, CD 19, heterogenous cCD 22, cytoplasmic IgM and negative for CD34 and terminal deoxynucleotidyl transferase (TdT). A cerebral spinal fluid (CSF) analysis was sent for and it showed no intracranial extension. Computed tomographic (CT) scan images of the brain revealed multiple, well-defined enhanced lesions at the frontal scalp with the largest lesion at the anterior glabella. The lesion extended into the bilateral orbital cavities and caused minimal lateral displacement of the bilateral orbits. Otherwise, the globes were normal (Figure 5). A repeated computed tomography (CT) scan of the brain showed resolved frontal swelling.

**Figure 6:**
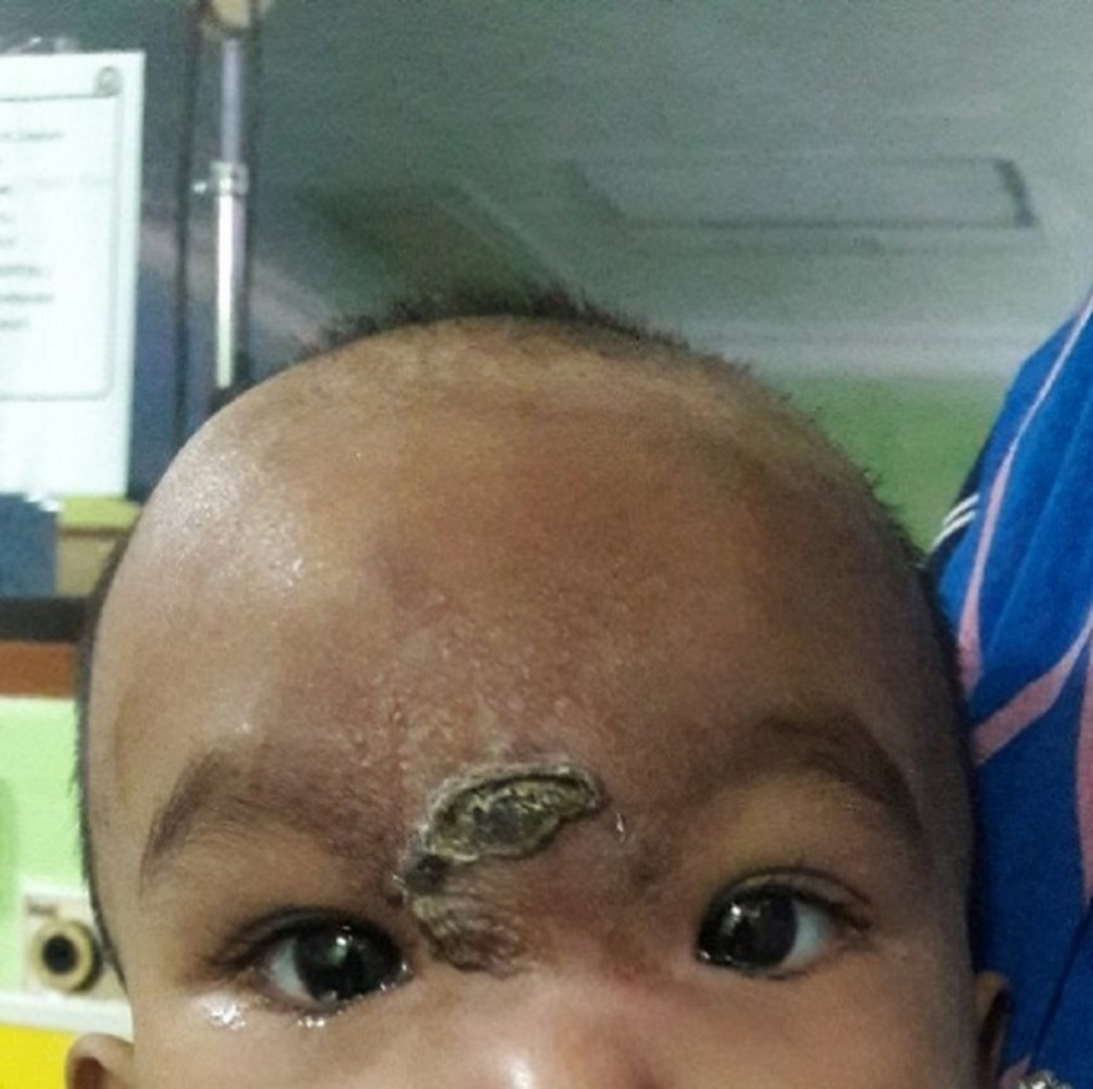
Significant reduction of the forehead swelling after chemotherapy.

Chemotherapy was commenced based on the United Kingdom (UK) Acute Lymphoblastic Lymphoma Protocols 97/99 (regime B: high risk). She attained remission of the disease with significantly reduced frontal swelling after the induction phase of chemotherapy (Figure 6). Currently, the patient is still under pediatric oncology follow-up and on the maintenance phase of chemotherapy.

## Discussion

Acute lymphoblastic lymphoma is a malignant disease of bone marrow that occurs in both children and adults. The incidence peaks between ages two and five years [2]. This type of lymphoma develops more frequently in boys than in girls with a male : female ratio of 55% : 45% [1].

Acute lymphoblastic lymphoma can be derived from B-cell or T-cell type of lineage [1]. In this case, the patient was diagnosed with B-lineage acute lymphoblastic lymphoma (ALL). It was supported by the full blood picture (FBP), bone marrow aspiration (BMA), and flow cytometry reports. In B-lineage ALL, the most important markers for diagnosis, differential diagnosis, and subclassification are CD19, CD20, CD22, CD24, and CD79a. The earliest B-lineage markers are CD19, CD22 (membrane and cytoplasm), and CD79a. Our patient’s flow cytometry result showed positivity for CD79a, HLA-DR, CD 10, CD 19, heterogenous cCD 22, and cIgM. Flow cytometry analysis represents the diagnostic gold standard for both the identification of cell lineage and the definition of subset [3].

The common presentation of acute lymphoblastic lymphoma is related to the suppression of hematopoiesis by leukemic cells, such as pallor, petechia, ecchymosis in skin and mucus membranes or infection due to neutropenia [1]. Growing skeleton is also an important site for proliferation of leukemic cells leading to limping gait and arthritis symptoms [4]. Few cases of acute lymphoblastic lymphoma with bony involvement and its radiographic finding have been described before, with the skull being an uncommon site of involvement [5]. Most of the cases with bony involvement presented with musculoskeletal pain, swelling and joint effusion, and ended with functional impairment [6]. Our patient presented with unique and rare symptoms. She developed a progressively increasing frontal swelling with bilateral upper eyelid involvement prior to the diagnosis of lymphoma. Radiological imaging showed multiple, well-defined enhanced lesions at the frontal scalp with the largest lesion at the anterior glabella. The lesion extended into the bilateral orbital cavities and caused minimal lateral displacement of the bilateral orbits. Otherwise, the bilateral globes were normal. Some authors have reported the case of acute lymphoblastic lymphoma in children who presented with proptosis due to orbital mass [7]. Others have reported a case of a child with acute myeloid leukaemia (AML) who presented with bitemporal swelling and proptosis [8].

The survival rate of childhood lymphoma is approaching 90% [9] with B-acute lymphoblastic lymphoma lineage and has a higher cure rate compared to T-acute lymphoblastic leukemia lineage [10]. The treatment usually starts based on a risk stratification, which is low, moderate, or high risk group [10]. Risk stratification is important in order to reduce toxicity in low risk group and to ensure adequate treatment for the high risk group. Treatment involves multidrug regimens to avoid the development of resistance. The four major treatment components are induction phase, consolidation phase, maintenance phase, and therapy directed against the central nervous system involvement [10].

Our patient was started on chemotherapy based on the United Kingdom Acute Lymphoblastic Lymphoma 97/99 Regime B (high risk of relapse). She was placed in the high risk group due to her age, initial white cell count more than 50 x 109/l, and the presence of extramedullary disease at diagnosis evidenced by cutaneous scalp lesions and hepatosplenomegaly. The Pediatric Oncology Group (POG) and Children's Cancer Group (CCG) adopted a common set of risk criteria acknowledged by the National Cancer Institute (NCI). The NCI criteria are based on factors that have international acceptance and are reproducible: age, initial white blood cell count, and the presence of extramedullary disease at diagnosis [11].

Our patient achieved remission of the disease after the induction phase of chemotherapy. This was evidenced by normalization of the full blood picture sample and bone marrow aspiration after four weeks of the induction phase. The forehead swelling was also significantly reduced. Currently, the patient is on the maintenance phase of chemotherapy. A patient in the low risk category has a four-year event-free survival (EFS) rate of approximately 80%. This includes patients aged one to nine years and with a white blood cell count at diagnosis less than 50 x 109/l. Whereas, a high-risk acute lymphoblastic lymphoma patient has a four-year event-free survival rate of approximately 65% [12].

## Conclusions

Acute lymphoblastic lymphoma should be kept in the differential diagnosis of a child who presents with extensive forehead swelling. Rapid and timely investigation and prompt treatment are important as ocular involvement of acute lymphoblastic lymphoma signifies poor prognosis. Early diagnosis and treatment can save and prolong the life of such patients.
